# PTBP1 enhances miR-101-guided AGO2 targeting to *MCL1* and promotes miR-101-induced apoptosis

**DOI:** 10.1038/s41419-018-0551-8

**Published:** 2018-05-10

**Authors:** Jia Cui, William J. Placzek

**Affiliations:** Department of Biochemistry and Molecular Genetics, The University of Alabama at Birmingham, Birmingham, AL 35294 USA

## Abstract

Myeloid cell leukemia 1 (MCL1) is a key anti-apoptotic protein belonging to the BCL-2 protein family. To preserve normal cellular homeostasis, cells must maintain strict control over MCL1 expression. Overexpression of MCL1 has been identified as a key contributor to tumorigenesis, and further enables resistance to a number of anti-cancer chemotherapies. Thus, there is an ongoing interest to develop selective MCL1 inhibitors. In order to better target MCL1, it is essential to understand the molecular mechanisms that regulate MCL1 expression in cells. While MCL1 expression is tightly controlled by multiple mechanisms, the post-transcriptional regulation of *MCL1* mRNA is poorly studied. Our previous work identified that polypyrimidine tract binding protein 1 (PTBP1) binds to *MCL1* mRNA and represses MCL1 expression by destabilizing *MCL1* mRNA. In this report, we show that PTBP1 modulates MCL1 expression by regulating the microRNA (miRNA) direction of the miRNA-induced silencing complex (miRISC) to *MCL1*. We demonstrate that PTBP1 enhances miR-101-guided AGO2 interaction with *MCL1*, thereby regulating miR-101-induced apoptosis and clonogenic cell survival inhibition in cells. Taken together, not only do these studies expand our understanding on the regulation of MCL1, they also demonstrate that PTBP1 and miRNAs can function cooperatively on a shared target mRNA.

## Introduction

Myeloid cell leukemia 1 (MCL1), an anti-apoptotic member of the BCL-2 protein family, is a key regulator of survival that inhibits intrinsic apoptosis or programmed cell death^1^. MCL1 functions as an anti-apoptotic regulator in two roles. Firstly, MCL1 binds to and suppresses the oligomerization of the pro-apoptotic BCL-2 proteins, BAK and BAX, thus preventing mitochondrial outer membrane permeabilization, cytochrome c release, and activation of downstream caspases^1^. Secondly, MCL1 directly interacts with the activator BH3-only proteins such as BID, BIM, and PUMA thereby blocking their ability to initiate BAK and BAX oligomerization^1,2^. Because of these roles, strict regulation of MCL1 expression is essential for normal cellular homeostasis and physiology including neuronal differentiation^3^ and blood cell maturation^4^. Overexpression of MCL1 is common in human cancers due to its pro-survival effect^5,6^ and has been reported to contribute to both tumorigenesis and resistance to multiple anti-cancer chemotherapeutics including anti-tubulin drugs^7,8^, platinum-containing compounds^9^, and therapeutics targeting other anti-apoptotic BCL-2 family members^10,11^. Due to its central positioning in cancer survival, there is an ongoing focus to develop anti-cancer therapies targeting MCL1, including small molecule inhibitors^12–14^, stapled peptides^15,16^, antisense oligonucleotides^17^, and reversible covalent inhibitors^18^.

While development of MCL1-targeted inhibitors is underway, it is important to understand the mechanisms that regulate MCL1 expression in cells to aid in better targeting of MCL1 and to avoid MCL1-mediated drug resistance. MCL1 expression is tightly controlled by multiple transcriptional, post-transcriptional, translational, and post-translational mechanisms^1^. The presence of a long 3′ untranslated region (3′-UTR) of ~2.8 kb and a short turnover time of ~1.5 h^8^ are indicative that there is a complex regulatory system to control *MCL1* mRNA. In cells, the 3′-UTR of an mRNA is a common regulatory region that associates with RNA binding proteins (RBPs) and microRNAs (miRNAs) that control the mRNA’s stability, localization, and translation^19^. Studies of the post-transcriptional regulators of *MCL1* mRNA have identified multiple validated and putative targeting miRNAs, e.g., miR-29a^20,21^, miR-101^22–24^, miR-125b^25^, miR-320^26^, miR-361 (predicted by microRNA.org^27^) etc. miRNAs are single-stranded non-coding RNAs of ~22 nucleotides that bind to the 3′-UTR and typically repress gene expression by either suppressing translation or activating degradation of the mRNA by mediating the binding of the miRNA-induced silencing complex (miRISC)^28^. miRNA activity has been shown to be strongly impacted by the conserved pairing of a target mRNA to the 5′ region of the miRNA centered on nucleotides 2–7 (miRNA seed region), as well as the number of additional interactions the target mRNA has with the 3′ nucleotide sequence, upstream of the seed^29^. As part of the miRISC, a miRNA couples with Argonaute protein (AGO) and serves to guide and mediate the binding of miRISC with target mRNA^30^. In mammals, only one of the AGO family members (AGO2) is endonuclease active and can process the cleavage of its target mRNA^31^. Previous studies have shown that these *MCL1*-targeting miRNAs suppress MCL1 expression, thereby inducing apoptosis, repressing cancer progression and development, and increasing drug sensitivity in different types of cancers such as lymphoma, osteosarcoma, hepatocellular carcinoma, triple negative breast cancer, endometrial cancer, and cervical cancer, etc^20–26^. Yet, limited studies have been done to determine the corresponding protein regulators of *MCL1* mRNA, and none to characterize the interplay between *MCL1*-targeting RBPs and *MCL1*-targeting miRNAs.

RNA-binding proteins play key roles in the post-transcriptional control of gene expression by regulating RNA metabolism, alternative splicing, stability, localization, and transportation in cells^32^. The interplay between miRNAs and RBPs has been shown to have a global effect on miRNA processing and activity, thereby shaping the miRNA-mediated gene repression post-transcriptionally^28^. Polypyrimidine tract binding protein 1 (PTBP1) or heterogeneous nuclear ribonucleoprotein I (hnRNP I) is a RBP belonging to the hnRNP family. PTBP1 interacts with RNA through four RNA recognition motifs (RRMs) that provide separate nucleotide specificity^33^. A number of studies have reported that PTBP1 is involved in diverse processes in RNA processing and metabolism^8,34–38^. Genome-wide mRNA decay analysis shows that the stability of hundreds of genes is significantly impacted after PTBP1 knockdown^37^.

Our previous work identified *MCL1* as a novel target of PTBP1^8^. We reported that PTBP1 binds to *MCL1* mRNA and regulate MCL1 expression by modulating *MCL1* mRNA stability^8^. Further, we demonstrated that the pro-survival effect of PTBP1 knockdown is largely due to the release of its control over MCL1^8^. However, the mechanisms by which *MCL1* mRNA was regulated by PTBP1 post-transcriptionally were not determined. In this report, we reveal that PTBP1 regulates miR-101-mediated AGO2 association with *MCL1*. Specifically, we identify that PTBP1 enhances miR-101-guided AGO2 targeting to *MCL1* and regulates miR-101-mediated apoptosis and clonogenic cell survival inhibition in cells.

## Results

### The effects of PTBP1 on *MCL1*-targeting miRNA expression

Based on our previous analysis of PTBP1 Crosslinking-immunoprecipitation Sequencing (CLIP-seq), which identified multiple binding sites in the 3′-UTR of *MCL1*^8^, we hypothesized that PTBP1 regulates MCL1 expression via the miRNAs/miRISC complex. Before exploring this hypothesis, we first sought to determine the impact that PTBP1 has on miRNA expression to ensure that the effect of PTBP1 knockdown on MCL1 is not simply due to a decrease in expression of *MCL1*-targeting miRNA. We therefore used the miRNA-specific TaqMan assays to assess the expression levels of five *MCL1*-targeting miRNAs (miR-29a, miR-101, miR-125b, miR-320, and miR-361)^20–26^, both in siControl and siPTBP1-treated H1299 (Supplementary Figure 1A) and PC3 (Supplementary Figure 1B) cells. We chose these cancer cell lines because they have been consistently studied in the BCL-2 field and were used in our initial characterization of PTBP1’s impact on MCL1^8^. In both the cell lines, we observed only a slight increase or no change in the expression levels of these five *MCL1*-targeting miRNAs upon PTBP1 knockdown (Supplementary Figure 1). This indicates that the increase of MCL1 by PTBP1 knockdown is not due to a corresponding decrease in the production of the *MCL1*-targeting miRNAs.

### AGO2 association with *MCL1* mRNA 3′-UTR

**Fig. 1:**
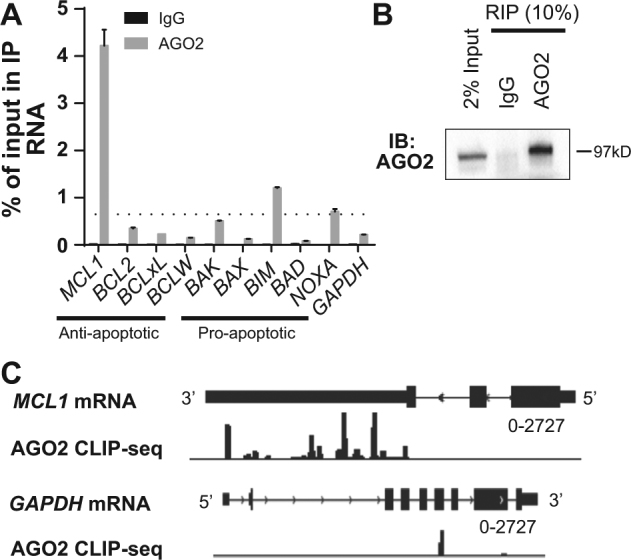
AGO2 binds to *MCL1*. **a** AGO2 association with BCL-2 family members and *GAPDH* mRNA in PC3 cells was assessed through RNA immunoprecipitation (RIP) with control IgG or AGO2 antibodies. The amount of RNA binding to AGO2 or IgG were quantified as percentage of input in IP by RT-qPCR. *GAPDH* RNA was used as a negative control. The dotted line depicts the cutoff of 3× the *GAPDH* IP level. **b** Western blot assessing AGO2 protein immunoprecipitation by AGO2 antibody in RIP. **c** The CLIP-seq analysis of AGO2 binding events in HELA cells are mapped on the *MCL1* and *GAPDH* genes (GSM1048187).

Our previous data demonstrated that PTBP1 silencing stabilizes *MCL1* mRNA^8^. mRNA decay is a complex process that is controlled by an interplay of multiple RBPs and miRNAs. As part of this, miRNAs facilitate sequence-specific targeting of AGO2 containing miRISC complex to target mRNAs^30^. In the miRISC, catalytically active AGO2 controls mRNA stability^31^. To further examine the involvement of the miRISC complex in the regulation of MCL1 by PTBP1, we next investigated the possible interaction between AGO2 and *MCL1* mRNA. First, we assessed AGO2 association with *MCL1* mRNA using RNA immunoprecipitation (RIP) with an AGO2 antibody or IgG control antibody, followed by real-time quantitative PCR (RT-qPCR). We observed that AGO2 interacted with *MCL1* mRNA in PC3 cells (Fig. 1). As shown in Figure 1a, b, *MCL1* was the predominantly enriched mRNA among the nine BCL-2 family members, with a ~300-fold increase in AGO2 RIP samples, compared with IgG RIP samples (Fig. 1b). To control for non-specific mRNA association, we also assessed *GAPDH* mRNA as a negative control and observed a significantly smaller enrichment by AGO2 RIP (Fig. 1a). Secondly, in order to map the binding sites on *MCL1*, we analyzed AGO2 binding to *MCL1* mRNA in HELA cells using available CLIP-seq data (GSM1048187)^37^. The AGO2 CLIP-seq identified multiple AGO2 binding sites within the 3′-UTR of *MCL1* mRNA (Fig. 1c). Both techniques demonstrated AGO2 association with *MCL1* mRNA in cells.

### Knockdown of AGO2 upregulates MCL1

**Fig. 2:**
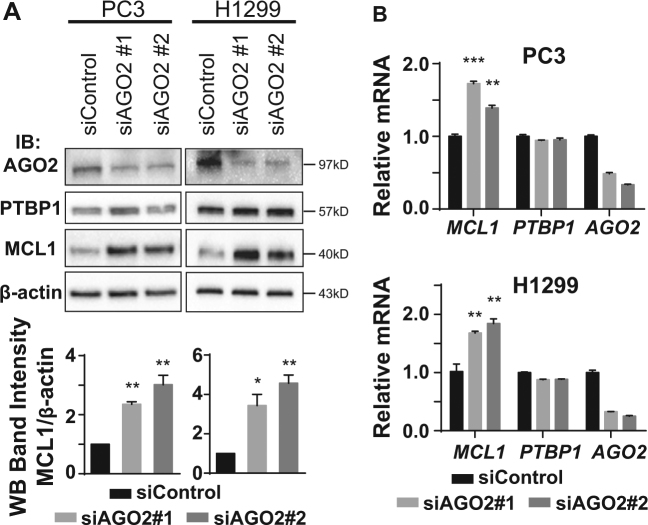
Knockdown of AGO2 upregulates MCL1. PC3 and H1299 cells were transfected with either siControl or one of two individual siRNAs targeting AGO2 (siAGO2#1 and siAGO2#2) for 48 h. **a** AGO2, PTBP1, MCL1, and β-actin levels were detected by western blotting. Relative MCL1 band intensity was quantified and normalized against β-actin. **b** Relative *MCL1*, *PTBP1*, and *AGO2* mRNA levels were quantified by RT-qPCR using *ACTB* as the internal control. Data is shown as mean ± SEM, *n* = 3. The statistical significance was determined using unpaired Student’s *t*-test where *p < 0.05; **p < 0.01; ***p < 0.001.

**Fig. 3:**
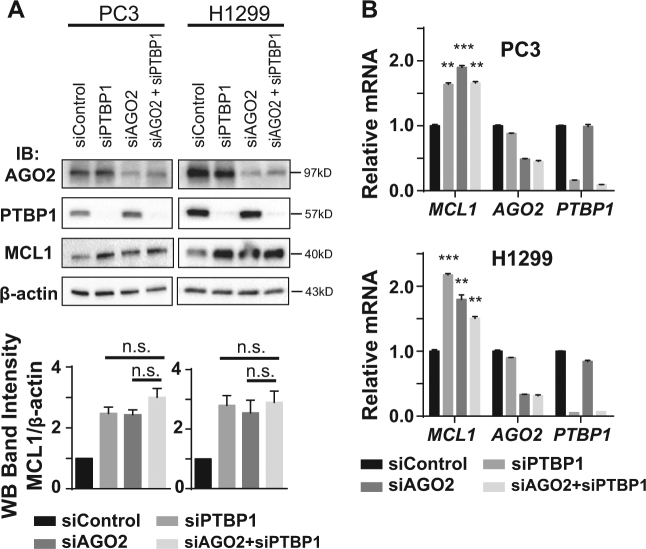
PTBP1 and AGO2 regulate MCL1 expression interdependently. PC3 and H1299 cells were transfected with siControl, siPTBP1, siAGO2, or siAGO2+siPTBP1 for 48 h. **a** AGO2, PTBP1, MCL1, and β-actin levels were detected by western blotting. Relative MCL1 band intensity was quantified and normalized against β-actin. **b** Relative *MCL1*, *AGO2, PTBP1* RNA levels were quantified by RT-qPCR using *ACTB* as the internal control. For all panels, data is shown as mean ± SEM, *n* = 3. The statistical significance was determined using unpaired Student’s *t*-test where n.s. (not significant) *p* > 0.05; **p < 0.01; ***p < 0.001.

The interaction between AGO2 and *MCL1* mRNA (Fig. 1) suggests that AGO2 plays a role in regulating MCL1 expression. We next transiently knocked down AGO2 using two individual siRNAs targeting *AGO2* mRNA (siAGO2#1 and siAGO2#2) for 48 h, and assessed the resulting effect on MCL1 protein levels by western blotting (Fig. 2a) and *MCL1* mRNA levels by RT-qPCR (Fig. 2b). We observed that AGO2 silencing elevated MCL1 expression significantly on both the protein and mRNA levels in a similar fashion, as we previously observed when we knocked down PTBP1^8^, which is recapitulated in Figure 3.

### PTBP1 and AGO2 regulate MCL1 expression interdependently

Since both PTBP1 silencing and AGO2 silencing increase MCL1 expression, we next assessed if PTBP1 and AGO2 regulate MCL1 expression on a shared signaling pathway. Cells were transfected with negative control siRNA (siControl), two individual siRNAs targeting either *PTBP1* (siPTBP1) or *AGO2* (siAGO2), or a mixture of the same amount of siAGO2 and siPTBP1 at a 1:1 ratio (siAGO2+siPTBP1) for 48 h. We observed comparable knockdown efficiency of AGO2 and PTBP1 in the dual knockdown group (siAGO2+siPTBP1) to that observed in either of the individual knockdown groups using siAGO2 or siPTBP1 alone (Fig. 3). Although siPTBP1 or siAGO2 alone increased MCL1 protein and mRNA levels significantly, siAGO2+siPTBP1 did not induce a significantly additive effect on MCL1 protein and mRNA levels, compared with siPTBP1 or siAGO2 alone (Fig. 3). This strongly suggests that PTBP1 and AGO2 regulate MCL1 expression interdependently.

### Knockdown of PTBP1 reduces AGO2 association with *MCL1*

**Fig. 4:**
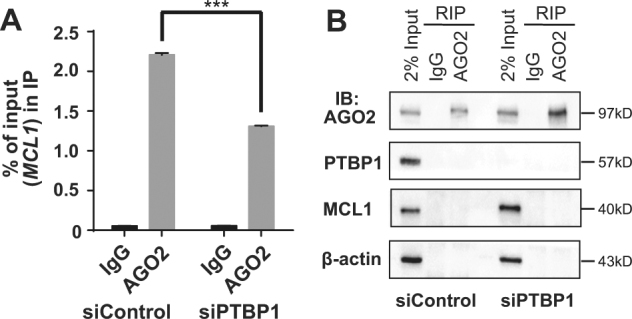
Knockdown of PTBP1 reduces AGO2 binding to *MCL1*. **a** RIP using IgG (control) or AGO2 antibody was performed in PC3 cells transfected with either siControl or siPTBP1 for 48 h. The amount of *MCL1* RNA binding to AGO2 was quantified as percentage of input in IP by RT-qPCR. **b** Immunoprecipitated AGO2, PTBP1, MCL1, and β-actin protein levels were assessed by western blotting. Data is shown as mean ± SEM, *n* = 3. The statistical significance was determined using unpaired Student’s *t*-test where ***p < 0.001.

**Fig. 5:**
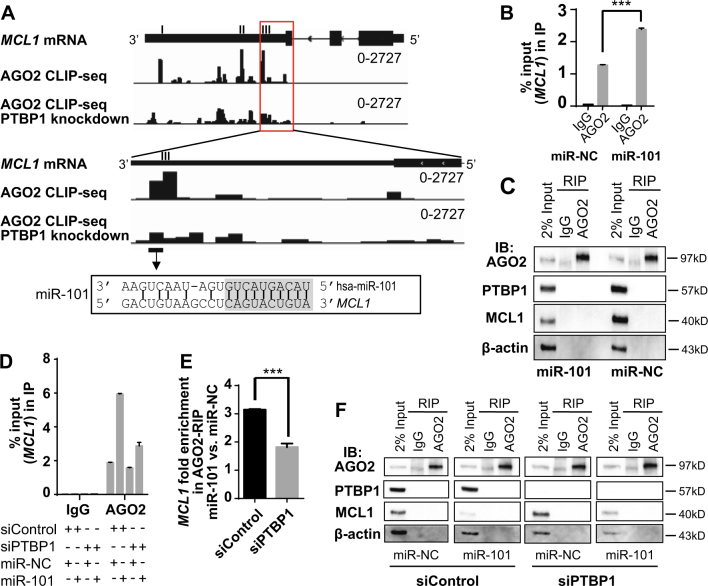
The interaction of *MCL1* with AGO2 is enhanced by miR-101, which is reduced by PTBP1 knockdown. **a** The CLIP-seq analysis of AGO2 binding events in control or PTBP1 knockdown HELA cells are mapped on *MCL1* RNA (GSM1048187 and GSM1048188). Three major peaks with decreased AGO2 binding signals upon PTBP1 knockdown were numbered I, II, and III. An *MCL1* mRNA sequence in peak III is highlighted with the miR-101 seed in gray shading. **b** RIP was performed in PC3 cells transfected with either negative control miRNA (miR-NC) or miR-101 mimic (miR-101) for 24 h. The amount of *MCL1* RNA binding to AGO2 (% of input in IP) was quantified by RT-qPCR. **d** H1299 cells were transfected with either siControl or siPTBP1 for 24 h, and then transfected with either miR-NC or miR-101 for another 24 h. RIP was then performed. The amount of *MCL1* RNA binding (% of input in IP) to AGO2 was quantified by RT-qPCR. **e** The effect of miR-101-induced AGO2 binding was calculated as *MCL1* fold enrichment in AGO2-RIP by miR-101 vs. miR-NC. **c**, **f** AGO2, PTBP1, MCL1, and β-actin protein levels in RIP were assessed by western blotting. Data is shown as mean ± SEM, *n* = 3. The statistical significance was determined using an unpaired Student’s *t*-test where *p < 0.05; **p < 0.01; ***p < 0.001.

To further test the working model that PTBP1 suppresses MCL1 levels by facilitating AGO2 targeting, we examined the effects of PTBP1 silencing on the interaction between AGO2 and *MCL1* mRNA. We performed an AGO2-RIP in PC3 cells and revealed that knockdown of PTBP1 by siRNAs for 48 h significantly reduced the amount of *MCL1* mRNA that binds to AGO2 (Fig. 4a). Control western blots from AGO2-RIP not only recapitulated our prior finding that PTBP1 knockdown increased MCL1 levels^8^, but failed to observe co-immunoprecipitation of PTBP1 with AGO2 (Fig. 4b), suggesting that these proteins do not form a complex to regulate MCL1 expression. To complement these studies, we analyzed AGO2-CLIP-seq in control and shPTBP1-treated HELA cells (GSM1048187 and GSM1048188)^37^ to map the regions of *MCL1* mRNA that underwent a change in binding to AGO2. In agreement with the AGO2-RIP analysis, PTBP1 silencing resulted in an overall decrease in AGO2 binding to *MCL1*. We further identified three target regions in *MCL1’s* 3′-UTR that underwent acute reduction in AGO2 binding following PTBP1 knockdown (Fig. 5a). These data suggest that PTBP1 regulates AGO2 association with *MCL1* mRNA, possibly targeting specific contact regions in *MCL1*.

### MiR-101 induces AGO2 binding to *MCL1*

Given the impact of PTBP1 on AGO2 association with *MCL1* and the necessity for miRNAs to guide AGO2 to target mRNAs, we next sought to determine which miRNAs are involved in PTBP1/AGO2 regulation of MCL1. As previously discussed, multiple miRNAs have been identified to target *MCL1*. Analysis of the AGO2-CLIP-seq data localized AGO2 binding to three primary sites (I, II, and III) that are strongly downregulated following PTBP1 knockdown (Fig. 5a). Examination of these three sites identified that site I and II do not contain any putative or validated miRNA seed sequences. We observed that site III, at chr1: 148,816,000-148,816,025, directly overlays with the miR-101 seed sequence (Fig. 5a). In Figure 5a, we zoom into the region of site III, highlighting the miR-101-binding site that falls directly under the AGO2-CLIP-seq identified region, and highlight the miR-101 seed sequence with gray shading. MiR-101 is a validated MCL1 suppressor^22–24^ that is commonly downregulated in cancer tissues^23,39,40^. Furthermore, miR-101 expression levels have been shown to negatively correlate with MCL1 expression levels in non-small cell lung cancer tissue, and this coupled reduction of miR-101 expression and increase of MCL1 expression are associated with a poorer clinical prognosis^39^. Due to the solid *in vitro*, *in vivo*, and clinical data supporting miR-101’s role in targeting MCL1, we next investigated the effect of miR-101 on the association between AGO2 and *MCL1* mRNA. To test this effect, we performed a RIP analysis in PC3 cells using the AGO2 antibody in the absence or presence of the overexpressed miR-101. We observed that overexpression of miR-101 in cells significantly increased the interaction of AGO2 with *MCL1* mRNA by ~twofold (Fig. 5b). The control western blots from the AGO2-RIP further confirmed that AGO2 protein was immunoprecipitated (Fig. 5c). Moreover, the 2% sample input lanes in Fig. 5c confirm that MCL1 protein levels are repressed upon miR-101 transfection, which agrees with the previous reports^22–24^.

### PTBP1 enhances miR-101-induced AGO2 interaction with *MCL1*

To test the hypothesis that PTBP1 enables miR-101 targeting of *MCL1*, we transfected H1299 cells with siControl or siPTBP1 firstly for 24 h and then transfected with miR-NC or miR-101 in the siControl or siPTBP1-treated cells for another 24 h. AGO2-RIP was performed to assess the amount of *MCL1* mRNA binding to AGO2 in the absence or presence of PTBP1 and miR-101 (Fig. 5d). The effect of PTBP1 on miR-101-induced AGO2 interaction with *MCL1* mRNA was calculated as the *MCL1* fold enrichment by miR-101, compared with miR-NC (Fig. 5e). In concordance with the prior studies of miR-101’s impact on *MCL1* mRNA (Fig. 5b), overexpression of miR-101 in siControl cells induced a significant increase (~3.2-fold) in AGO2 binding with *MCL1* mRNA, compared to miR-NC treated siControl cells (Fig. 5e). In comparison, siPTBP1 knockdown cells suppressed the impact of miR-101 transfection on AGO2 binding with *MCL1* mRNA to only a ~1.8-fold increase (Fig. 5e). The control western blots were performed to confirm that AGO2 protein was immunoprecipitated in each RIP reaction (Fig. 5f). Similar results were observed in PC3 cells (Supplementary Figure 2).

### MCL1 repression by miR-101 is inhibited by PTBP1 knockdown

**Fig. 6:**
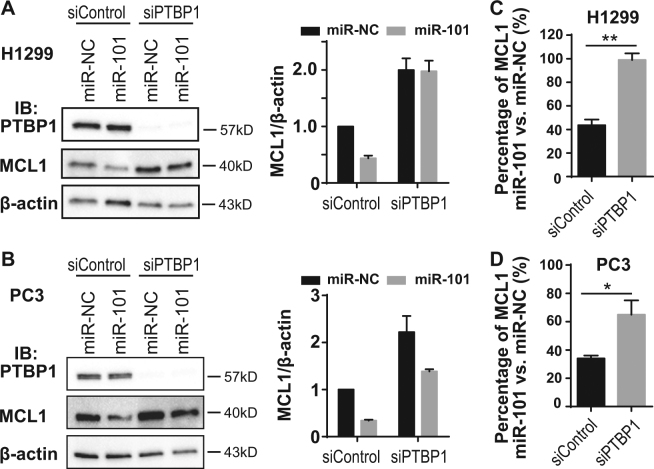
MiR-101’s repression of MCL1 expression is inhibited by PTBP1 knockdown. **a**, **b** H1299 and PC3 cells were transfected with siControl or siPTBP1 first for 24 h and then transfected with miR-NC or miR-101 for another 24 h. Total cell lysates were collected. PTBP1, MCL1, and β-actin levels were detected by western blotting. Relative MCL1 band intensity was quantified and normalized against β-actin. **c**, **d** The effect of miR-101 targeting MCL1 was calculated as the percentage of MCL1 upon miR-101 treatment, compared with miR-NC treatment. Data is shown as mean ± SEM, *n* = 3. The statistical significance was determined using unpaired Student’s *t*-test where *p < 0.05; **p < 0.01.

The inhibition of miR-101-induced AGO2 binding to *MCL1* mRNA by PTBP1 knockdown (Fig. 5) led to our hypothesis that the suppressive effect of miR-101 on MCL1 expression is regulated by PTBP1. To test this hypothesis, we sought to determine if loss of PTBP1 impacted miR-101 suppression of MCL1 expression. To do this, we transfected PC3 and H1299 cells with siControl or siPTBP1 for 24 h and then transfected with miR-NC or miR-101 for another 24 h. MCL1 protein levels were then analyzed by western blotting and relative MCL1 band intensity was quantified using Image Lab (Bio-rad Laboratories), with β-actin serving as the internal control (Fig. 6a, b). We calculated the suppressive effect of miR-101 on MCL1 expression as the percentage of MCL1 band intensity in miR-101-treated cells versus that in the miR-NC-treated cells (Fig. 6c, d). We observed that knockdown of PTBP1 in H1299 cells almost completely blocked (Fig. 6a,c), while in PC3 cells it partially rescued (Fig. 6b, d), miR-101’s repressive effect on MCL1. These data demonstrate that PTBP1 enhances, in a cell line-dependent manner, miR-101’s control over MCL1 expression.

### The PTBP1–AGO2/miR-101 axis specifically regulates MCL1

To determine if the outlined mechanism is specific in its regulation of MCL1 or if it broadly targets the BCL-2 family, we assessed the interaction of the central regulatory proteins with eight additional BCL-2 family proteins: BCL2, BCLxL, BCLW, BAK, BAX, NOXA, BIM, and BAD. Firstly, we assessed the association of AGO2 with each family member’s mRNA using RIP (Fig. 1a). We found that AGO2 interaction with *MCL1* is unique among the anti-apoptotic BCL-2 family members. We did identify a significant though 3× lower association of AGO2 with *BIM* and *NOXA,* compared to *MCL1*. From this, we conclude that AGO2 predominantly interacts with *MCL1*, compared with other BCL-2 family members. Secondly, we assessed the miR-101 targeting of the nine identified BCL-2 family members. While sequence analysis identifies that only *BIM* and *MCL1* contain a miR-101 seed sequence, we tested the impact of miR-101 on the expression of each of the BCL-2 family members in PC3 cells. Corresponding western blot analysis shows that only MCL1 exhibits a significant decrease in protein expression in miR-101-treated versus miR-NC-treated cells (Supplementary Figure 3). Thirdly, to characterize PTBP1 association with these nine family members, we reviewed PTBP1 CLIP-SEQ data for PTBP1’s mRNA association. This analysis suggests that *MCL1* is the predominant PTBP1 bound mRNA among the identified BCL-2 family members (supplementary Figure 4). Based on these three analyses, we expect that PTBP1-mediated post-transcriptional regulation via AGO2/miR-101 is a specific mechanism for controlling MCL1 expression in cells.

### PTBP1 silencing overcomes the impact of miR-101 on apoptosis and clonogenic cell survival

**Fig. 7:**
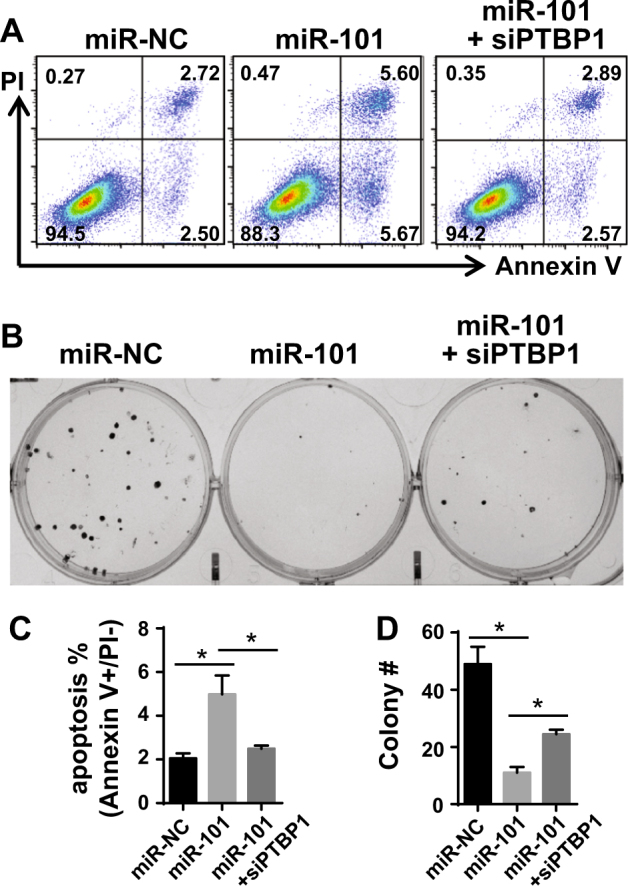
PTBP1 regulates miR-101’s function on apoptosis and clonogenic cell survival. H1299 cells were transfected with miR-NC, miR-101, or miR-101+siPTBP1. **a** After 48 h, cells were collected and stained with Annexin V/PI, followed by flow cytometry analysis. Annexin V+/PI− cells were considered apoptotic. **b** Colony formation assay of H1299 cells. Nine days after seeding, cells were fixed and stained with 0.5% crystal violet, and the number of colonies was counted. **c** Comparative bar graph depicting the observed apoptosis rate (%, Annexin V+/PI−) for each treatment in (**a**). **d** Statistical analysis of the colony number (#) between each group. Data is shown as mean ± SEM where *n* = 3 in (**c**) and *n* = 2 in (**d**). The statistical significance was determined using unpaired Student’s *t*-test where *p < 0.05.

Previous studies have identified that miR-101 is a pro-apoptotic miRNA, largely due to its downregulation of MCL1^22–24^. Likewise, our previous studies highlighted that PTBP1 silencing is anti-apoptotic as it upregulates MCL1^8^. These published findings, coupled with our current data demonstrating that PTBP1 regulates miR-101-induced AGO2 targeting to *MCL1* (Fig. 5) and miR-101’s repression of MCL1 expression (Fig. 6), led us to test if PTBP1 could regulate miR-101’s function on short-term apoptosis and long-term clonogenic cell survival. Apoptosis was assessed in H1299 cells by Annexin V/PI staining and flow cytometry analysis after 48 h of siRNA or miRNA transfection (Fig. 7a). In agreement with prior studies, miR-101 overexpression induced a ~twofold increase in apoptosis^22–24^. We observed that transfection of siPTBP1 in the miR-101-overexpressed cells rescued this effect, returning the percentage of apoptotic cells to the control level (Fig. 7c). These results suggest that the pro-apoptotic effects of miR-101 are PTBP1 dependent. Further, they support the cooperative function of PTBP1 and miR-101 on the shared target *– MCL1*.

**Fig. 8:**
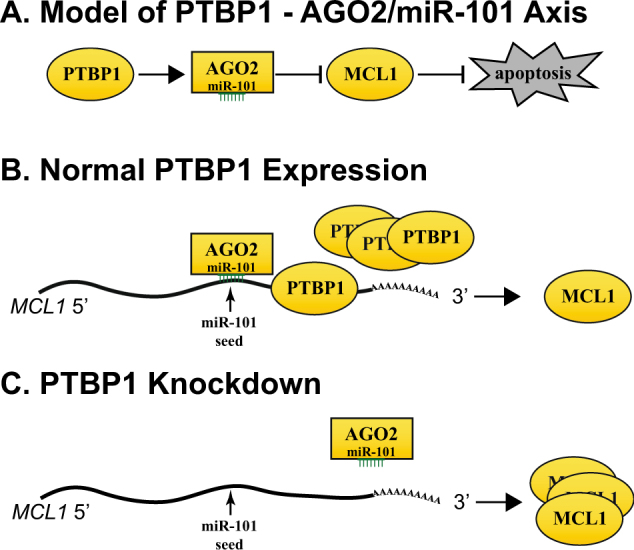
A proposed model of PTBP1 regulation of miR-101/AGO2 targeting of *MCL1*. **a** A summary diagram of the PTBP1–AGO2/miR-101 axis regulation of MCL1. **b** In the presence of normal PTBP1 expression, PTBP1 binds to *MCL1* and facilitates the association of AGO2/miR-101 with MCL1 mRNA, regulating MCL1 levels. **c** When PTBP1 expression is suppressed, AGO2/miR-101 interaction with *MCL1* is reduced. As a result, MCL1 expression increases.

We next sought to determine how PTBP1 knockdown affects the reproductive cell death of miR-101 treatment using a clonogenic survival assay^41^. H1299 cells were treated with siRNA or miRNA for 9 days and colony numbers (colony #) were counted using crystal violet staining (Fig. 7b). We observed an 80% reduction in clonogenic cell survival of H1299 cells following treatment with miR-101. Further, in line with our analysis on the impact of PTBP1 on miR-101 regulating MCL1 and apoptosis, we observed a partial rescue in siPTBP1+miR-101 treated cells (Fig. 7d). Taken together, our data suggest that PTBP1 enhances miR-101-guided AGO2 targeting to *MCL1*, thereby repressing MCL1 expression and increasing miR-101’s effects on apoptosis and clonogenic cell survival inhibition in the cells (Fig. 8a).

## Discussion

Post-transcriptional regulation is a delicate system that involves different processes and editing of both pre-mRNAs and mature mRNAs, *e.g.*, 5′ capping, 3′ polyadenylation, alternative splicing of the pre-mRNAs, degradation, and transportation control of the mature mRNAs etc^42^. Studying the basic mechanisms of post-transcriptional control is necessary to gain a comprehensive understanding of how gene expression is regulated at different levels and how aberrant post-transcriptional regulation is involved in multiple diseases, including cancer.

*MCL1* mRNA has a short turnover time of ~1.5 h. However, its long 3′-UTR of ~2.8 kb makes it difficult to untangle the involved molecular regulators. Therefore, the post-transcriptional regulation of *MCL1* mRNA has been poorly studied at a mechanistic level. Our work illustrates for the first time a detailed post-transcriptional regulation mechanism of *MCL1* mRNA by PTBP1 in cells. Based on the presented data and studies cited thus far, we propose a model in Figure 8 that when PTBP1 expression is high, PTBP1 binds to *MCL1* mRNA, which facilitates miR-101-loaded AGO2 interaction with *MCL1*, and thus MCL1 expression is suppressed (Fig. 8b). However, in the presence of low PTBP1 expression, as is seen during neuron^37,43^, muscle^44^, and cardiomyocyte^45^ differentiation, PTBP1-*MCL1* mRNA interaction decreases, resulting in masking of the miRNA-101 site on *MCL1* mRNA. As a result, *MCL1* mRNA is stabilized and MCL1 expression is then elevated (Fig. 8c).

*MCL1* mRNA has been reported to interact with various RBPs such as PTBP1^8^, CUGBP2^46^, and HuR^47^ and targeted by multiple miRNAs^20–26,38,48^. However, no studies have been performed to identify the interplay between RBPs and miRNAs on regulating MCL1 expression. Our current report is the first detailed study interrogating the crosstalk between a RBP (PTBP1) and a miRNA (miR-101) in regulating *MCL1* mRNA and apoptosis, and further sheds light on the complexity of the post-transcriptional regulation of *MCL1* mRNA.

Identification of the process or molecular entity that PTBP1 unmasks to facilitate miR-101 targeting of *MCL1* still remains to be identified. It is possible that PTBP1 binding changes the structure of *MCL1* mRNA or competes off other RBPs that inhibit miR-101/AGO2 binding, therefore unmasking the miR-101 recognition site. However, our preliminary analysis of the RNA fold of *MCL1* mRNA suggests that there is no secondary structure formed around the miR-101 seed sequence (unpublished data). Further studies are required to determine the secondary or tertiary structure of *MCL1* mRNA throughout its large 2.8 kb 3′-UTR. A detailed RNA footprinting analysis of the exact binding sites on *MCL1* by PTBP1 and its different RRMs is also necessary to completely understand PTBP1’s role in regulating MCL1 and apoptosis. As an initial example of the cooperative effects of PTBP1 and miR-101 on regulating *MCL1*, our report opens up the field to establish a network among different RBPs and miRNAs that regulate MCL1 expression post-transcriptionally. Further, it will be important to understand their corresponding impacts on apoptosis in both normal human physiology and diseases including cancers.

Since MCL1, PTBP1, and miR-101 are central regulators of various cellular processes, all have valuable therapeutic potentials in anti-cancer treatment. MCL1, as a key anti-apoptotic protein, is commonly overexpressed in cancers and selective MCL1 inhibitors have shown significant effects in in vivo and in vitro cancer models^12–18^. PTBP1, as a RBP, is a master regulator for RNA processing and metabolism. PTBP1 has differential impact on malignancy in a cell line-dependent manner^49^, but a number of studies have shown that PTBP1 is overexpressed in breast cancer^50^, colorectal cancer^51^, ovarian cancer^52^, and brain tumors^53,54^. Due to its global effect on regulating gene expression post-transcriptionally, PTBP1 inhibitors are also under development^55^. In addition, miRNA-based therapies have drawn great attention from different research groups and pharmaceutical companies with various miRNA mimics or inhibitors at preclinical, phase I, or phase II clinical stages^56^. MiR-101 is downregulated in multiple cancer types^23,40,57^ and plays critical roles in cancer development and progression including proliferation, apoptosis, invasion, motility, and colony formation^22–24^. Our model shows that these three factors do not work independently, but PTBP1 represses MCL1 expression by facilitating miR-101 targeting to *MCL1*. Therefore, while therapeutics acting on these three factors are still under development, it is necessary to consider the crosstalk between PTBP1, MCL1, and miR-101 in order to best deploy the resulting molecules for optimized impact in treating cancer patients.

## Material and methods

### Cell culture

PC3 and H1299 cells were grown in RPMI-1640 medium supplemented with 10% fetal bovine serum, 2.05 mM L-glutamine, and 100 units/ml penicillin and streptomycin (Life Technologies) in a humidified atmosphere with 5% CO_2_. Cells were authenticated using short tandem repeat profiling against published ATCC signatures.

### siRNA and miRNA transfections

Silencer^®^ Select siRNAs, negative control siRNA, mirVana^TM^ miRNA mimics, and negative control miRNA were purchased from Ambion (Carlsbad, CA, USA). The sequences of the Silencer^®^ Select siRNAs are: siPTBP1#1 (s11435) CAGUUUACCUGUUUUUAAAtt, siPTBP1#2 (s11436) GCAUCACGCUCUCGAAGCAtt, siAGO2#1 (19706) GGAGAGUUAACAGGGAAAUtt, and siAGO2#2 (133830) CGGCAGGAAGAAUCUAUACtt. The mature sequence of hsa-miR-101 mimic is UACAGUACUGUGAUAACUGAA. siRNA and miRNA transfections were performed using Lipofectamine RNA iMAX (Life Technologies, Carlsbad, CA, USA) reagent, following the manufacturer’s protocol. Cells were harvested for 24 or 48 h after transfection for analysis, as specified in text.

### TaqMan microRNA assay

Total transcriptome RNA were extracted by TRIzol reagent (Life Technologies) and purified by PureLink RNA Mini kit (Ambion), according to the TRIzol Plus total transcriptome isolation protocol. miRNAs were reverse transcribed using the TaqMan microRNA Reverse Transcription Kit (Life Technologies). qPCR was carried out using the TaqMan Universal PCR master mix II with UNG and TaqMan small RNA assay reagent (20×) for indicated miRNAs (Life Technologies) on the ViiA7 system (Applied Biosystems, Foster City, CA, USA).

### RNA extraction, reverse transcription, and qPCR

Total RNA was extracted by TRIzol reagent (Life Technologies) from fresh cells and purified using the PureLike RNA Mini kit (Ambion). Total RNA was treated with RNase-free DNase I (Thermo Scientific, Waltham, MA, USA) to remove the genomic DNA. Final RNA concentrations were determined using absorbance at 260 nm (A260) on a Nanodrop 2000c spectrophotometer with an A260/A280 ratio of ~2.1. Total RNA was reverse transcribed in 20 µl reactions using the qScript cDNA SuperMix (Quanta Biosciences, Beverly, MA, USA), according to the manufacturer’s protocol.

qPCR analysis was performed in 10 µl reactions, with 4 µl of the diluted cDNA, 5 µl of the Power SYBR Green Master Mix (Applied Biosystems), and 0.5 µl of each of the forward and reverse primers, at a final primer concentration of 250 nm. All qPCR reactions were performed in triplicate in MicroAmp Optical 384-well plates (Thermo Fisher) on the ViiA 7 system (Applied Biosystems). Amplification conditions were as follows: 10 min at 95 °C first, then 40 cycles of 10 s at 95 °C, and 1 min at 60 °C. Melting curves were generated in the end to confirm that the qPCR reactions produced single and specific products. Final products were run on agarose gel to confirm that the amplicons are of the correct size. The CT values of all non-template control reactions were below the detection limit. Data was analyzed using the comparative CT (∆∆CT) method using *ACTB* as an internal control in ViiA 7 RUO software and Excel, and exported to GraphPad Prism (GraphPad Software, La Jolla, CA, USA) for presentation. All qPCR data presented in figures are representative results of two or three biological replicates, as denoted in figure captions.

The following primers were used (5′–3′):

*MCL1* (NM_021960.4, amplicon 154 bp): (F) GGACATCAAAAACGAAGACG and (R) GCAGCTTTCTTGGTTTATGG; *BCL2* (NM_000633.2, amplicon 196 bp): (F) ATGTGTGTGGACAGCGTCAACC and (R) TGAGCAGAGTCTTCAGAGACAGCC; *BCLxL* (NM_138578.2,amplicon 85 bp): (F) GGTCGCATTGTGGCCTTT and (R) TCCGACTCACCAATACCTGCAT; *BCLW* (NM_004050.4, amplicon 231 bp): (F) GAGATGAGTTCGAGACCCG and (R) CCATCCACTCCTGCACTTG; *BAK* (NM_001188.3, amplicon 136 bp): (F) GGTTCTGGGTGTGGTTCTG and (R) AGGGAACAGAGAAGGCAAAG; *BAX* (NM_001291428.1, amplicon 137 bp): (F) GACATGTTTTCTGACGGCAAC and (R) AAGTCCAATGTCCAGCCC; *BIM* (NM_138621.4, amplicon 75 bp): (F) TCGGACTGAGAAACGCAAG and (R) CTCGGTCACACTCAGAACTTAC; *BAD* (NM_004322.3, amplicon 72 bp): (F) ACGTAACATCTTGTCCTCACAG and (R) GTCTTCCTGCTCACTCGG; *NOXA* (NM_021127.2, amplicon 123 bp): (F) GGAGATGCCTGGGAAGAAG and (R) TGCCGGAAGTTCAGTTTGTC; *PTBP1* (NM_002819.4, amplicon 123 bp): (F) ATTGTCCCAGATATAGCCGTTG and (R) GCTGTCATTTCCGTTTGCTG; *AGO2* (NM_002819.4, amplicon 148 bp)*:* (F) AAGGTGGAGATAACGCACTG and (R) TGTCCTTGAAATACTGGGCC; *ACTB* (NM_001101.4, amplicon 148 bp): (F) ACCTTCTACAATGAGCTGCG and (R) CCTGGATAGCAACGTACATGG.

### Protein extraction and western blotting

Cells were lysed by incubating with lysis buffer (Pierce) on ice for 10 min. The contents of lysis buffer are 25 mM Tris-HCl pH 7.4, 150 mM NaCl, 1% NP-40, 1 mM EDTA, 5% glycerol supplemented with Halt protease inhibitor cocktail (Thermo Scientific). Following lysis, the samples were cleared by centrifugation at 14,000×*g*, and the final protein concentration was determined by the Pierce BCA Protein Assay Kit (Thermo Scientific).

Equal amounts of cell lysates were resolved by SDS-PAGE and transferred to PDVF membrane. Membranes were incubated with anti-AGO2 antibody (RN003M, MBL International, Woburn, MA, USA), anti-PTBP1 antibody (RN011P, MBL International), anti-MCL1 antibody (D35A5, Cell Signaling), and anti-β-actin antibody (PA1-21167, Pierce/Thermo Fisher) at a dilution of 1:1000 at 4 °C overnight. On the next day, after wash, the membranes were incubated with HPR-conjugated secondary antibodies (Cell Signaling, Danvers, MA, USA) at room temperature for 1 h and detected with ECL2 reagents (Pierce) on the Bio-Rad ChemiDoc MP imaging system (Bio-Rad Laboratories, Hercules, CA). Band intensities were quantified using the Bio-Rad Image Lab software.

### RNA immunoprecipitation

RNA IP was performed using the RIP-Assay Kit for microRNA (MBL International). In brief, anti-AGO2 antibody (RN003M, MBL International) or mouse IgG control (Life Technologies) were immobilized with protein G magnetic beads (Dynabeads, Life Technologies), with rotation at 4 °C for 4 h. The cell extracts were precleared with protein G magnetic beads (Dynabeads) at 4 °C for 1 h. The precleared lysates were then incubated with the antibody-immobilized protein G beads at 4 °C overnight. After washing, the immunoprecipitated proteins were eluted by boiling with Laemmli sample buffer at 95 °C for 10 min. The immunoprecipitated RNAs were isolated using the two-step method outlined in the manufacturer’s manual. RNA was reserve transcribed and the target RNA was assessed by qPCR, as outlined above.

### Annexin V/PI staining and flow cytometry

H1299 cells were transfected with siRNAs and/or miRNAs in 6-well plates. After 48 h, the cells were trypsinized and counted with trypan blue. A total of 1 × 10^5^ cells were incubated with Annexin V-FITC and propidium iodide^5^ in Annexin V binding buffer (BD Biosciences, San Jose, CA, USA) at room temperature for 15 min. Then cells were run on BD LSRFortessa FACS, and 50,000 events were collected for each sample. Data was analyzed using FlowJo V10 (FlowJo, LLC, Ashland, OR, USA). For analysis, the cells sorted as Annexin V+/PI− were classified apoptotic.

### Colony formation assay

As outlined above, H1299 cells were transfected with siRNAs and/or miRNAs, and 1000 cells were seeded per well in 6-well plates. After 48 h, cells were switched to a complete RPMI-1640 medium. Then the cells were incubated in a CO_2_ incubator at 37 °C for another one week. After a total of 9 days, the cells were fixed with methanol at room temperature for 30 min and stained with 0.5% crystal violent (Sigma, St. Louis, MO, USA) with 25% methanol at room temperature for 1 h. The plates were then washed under running water and dried. The number of colonies per well were counted. A colony was defined as a group of at least 50 cells under the microscope.

### Statistical analysis

GraphPad Prism and Microsoft Excel were used for the statistical analysis. Statistical significance was considered using the unpaired two-tailed Student’s *t*-test for all analysis where *p < 0.05, **p < 0.01, ***p < 0.001.

## Electronic supplementary material

Supplementary Figure S1: PTBP1 knockdown does not decrease the production of MCL1-targeting miRNAs

Supplementary Figure S2: MiR-101 induced AGO2 interaction with MCL1 is reduced by PTBP1 knockdown in PC3 cells

Supplementary Figure S3: MCL1 is the only target of miR-101 among the BCL2 family

Supplementary Figure S4: MCL1 is the predominant BCL2 family mRNA bound by PTBP1
