# Supplementary material for: PTBP1 enhances miR-101-guided AGO2 targeting to MCL1 and promotes miR-101-induced apoptosis
Source: Cell Death Dis. 2018 May 10;9(5):552. doi: 10.1038/s41419-018-0551-8 (PMC5945587; doi:10.1038/s41419-018-0551-8)
Supplement: Supplementary file 1 — Supplementary Figure S1: PTBP1 knockdown does not decrease the production of MCL1-targeting miRNAs [file 41419_2018_551_MOESM1_ESM.pdf]

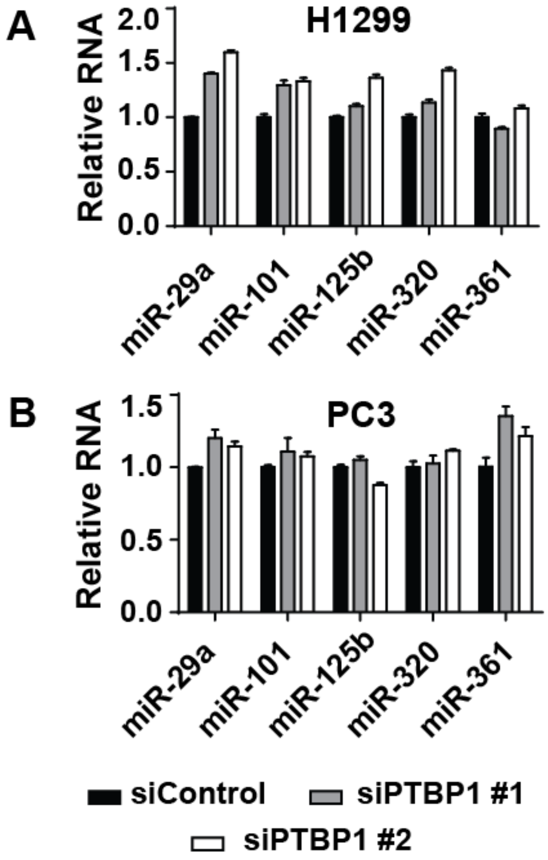

Supplementary Figure S1: **PTBP1 knockdown does not decrease the production of *MCL1*-targeting miRNAs**. (A and B) H1299 and PC3 cells were transfected with either siRNA negative control (siControl) or one of two individual siRNAs targeting PTBP1 (siPTBP1#1 and siPTBP1#2) for 48 h. Relative miRNA expression levels were quantified by TaqMan MicroRNA Assays and normalized against U18. Data is shown as mean ± SEM, n=3.
