# Supplementary material for: PTBP1 enhances miR-101-guided AGO2 targeting to MCL1 and promotes miR-101-induced apoptosis
Source: Cell Death Dis. 2018 May 10;9(5):552. doi: 10.1038/s41419-018-0551-8 (PMC5945587; doi:10.1038/s41419-018-0551-8)
Supplement: Supplementary file 2 — Supplementary Figure S2: MiR-101 induced AGO2 interaction with MCL1 is reduced by PTBP1 knockdown in PC3 cells [file 41419_2018_551_MOESM2_ESM.pdf]

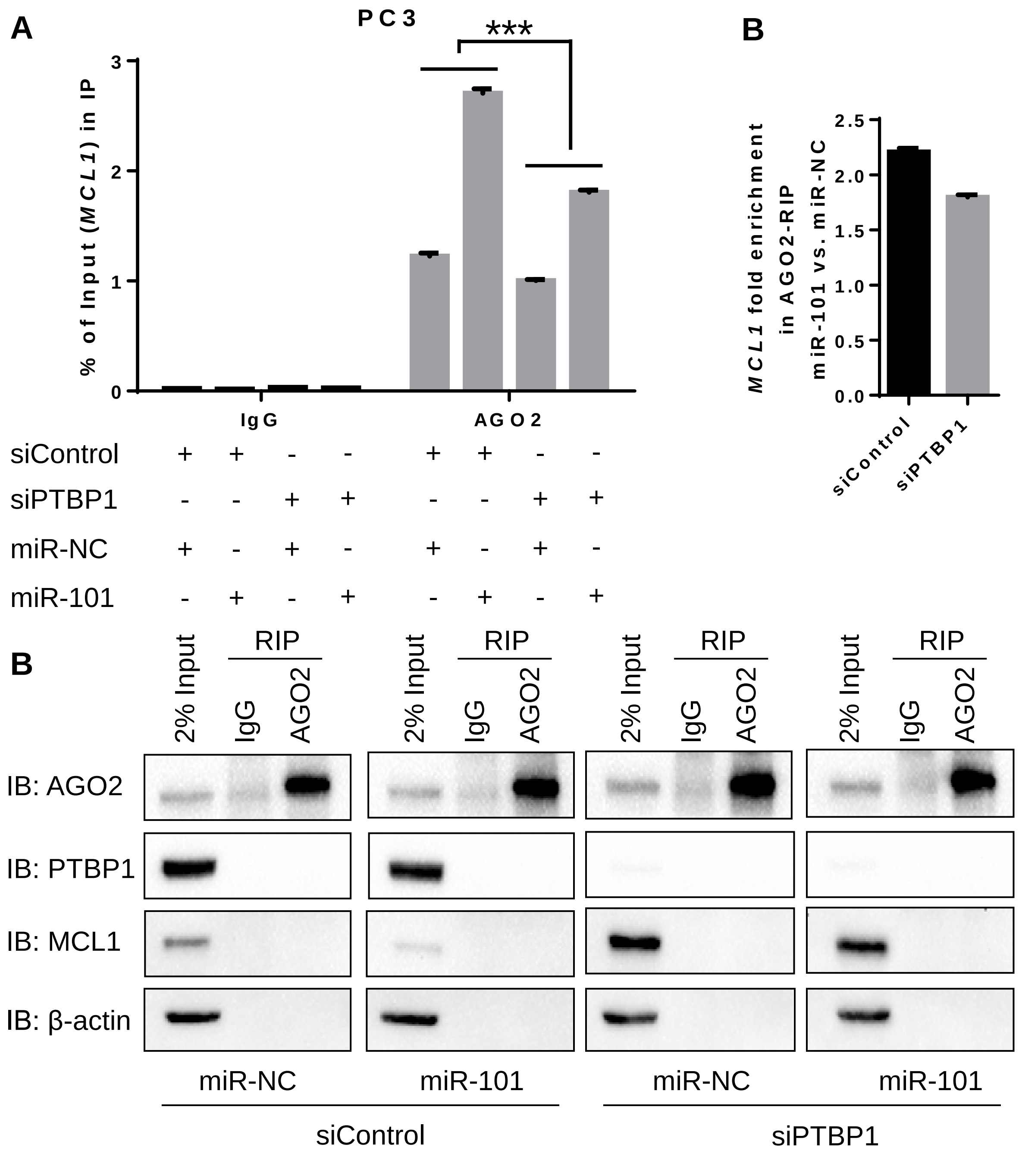

Supplementary Figure S2: **MiR-101 induced AGO2 interaction with *MCL1* is reduced by PTBP1 knockdown in PC3 cells**. (A) PC3 cells were transfected with either siControl or siPTBP1 for 24 h and then transfected with either miR-NC or miR-101 for another 24 h. RIP was then performed. The amount of *MCL1* RNA binding (% of input in IP) to AGO2 was quantified by RT-qPCR. (B) The effect of miR-101-induced AGO2 binding was calculated as MCL1 fold enrichment in AGO2-RIP by miR-101 vs. miR-NC. (C) AGO2, PTBP1, MCL1, and β-actin protein levels in RIP were assessed by western blotting. Data is shown as mean ± SEM, n=3. The statistical significance was determined using an unpaired Student's t-test where ***$p<0.001$.
