# Supplementary material for: PTBP1 enhances miR-101-guided AGO2 targeting to MCL1 and promotes miR-101-induced apoptosis
Source: Cell Death Dis. 2018 May 10;9(5):552. doi: 10.1038/s41419-018-0551-8 (PMC5945587; doi:10.1038/s41419-018-0551-8)
Supplement: Supplementary file 3 — Supplementary Figure S3: MCL1 is the only target of miR-101 among the BCL2 family [file 41419_2018_551_MOESM3_ESM.pdf]

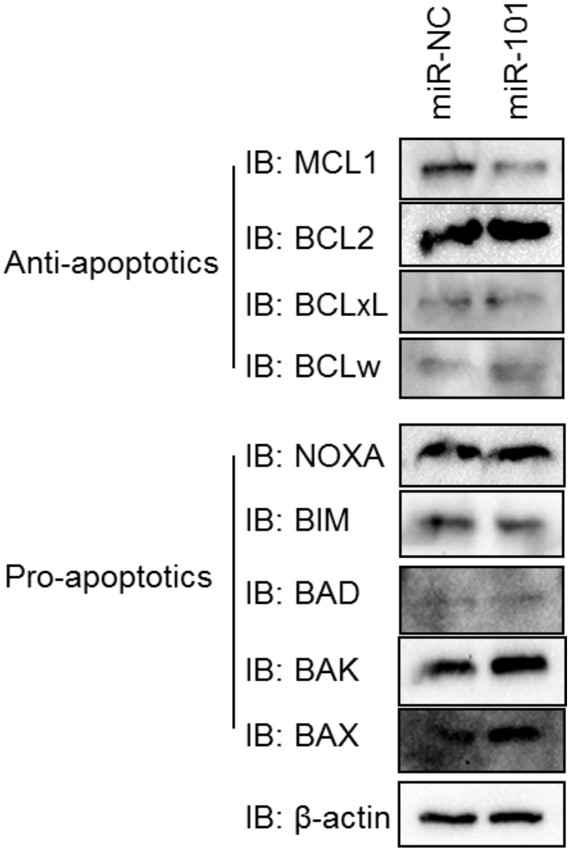

Supplementary Figure S3: **MCL1 is the only target of miR-101 among the BCL2 family**. PC3 cells were transfected with either miR-NC or miR-101 mimics for 24 h. Western blotting were performed to analyze the expression levels of different BCL2 family proteins. β-actin was used as the loading control.
