# Supplementary material for: PTBP1 enhances miR-101-guided AGO2 targeting to MCL1 and promotes miR-101-induced apoptosis
Source: Cell Death Dis. 2018 May 10;9(5):552. doi: 10.1038/s41419-018-0551-8 (PMC5945587; doi:10.1038/s41419-018-0551-8)
Supplement: Supplementary file 4 — Supplementary Figure S4: MCL1 is the predominant BCL2 family mRNA bound by PTBP1 [file 41419_2018_551_MOESM4_ESM.pdf]

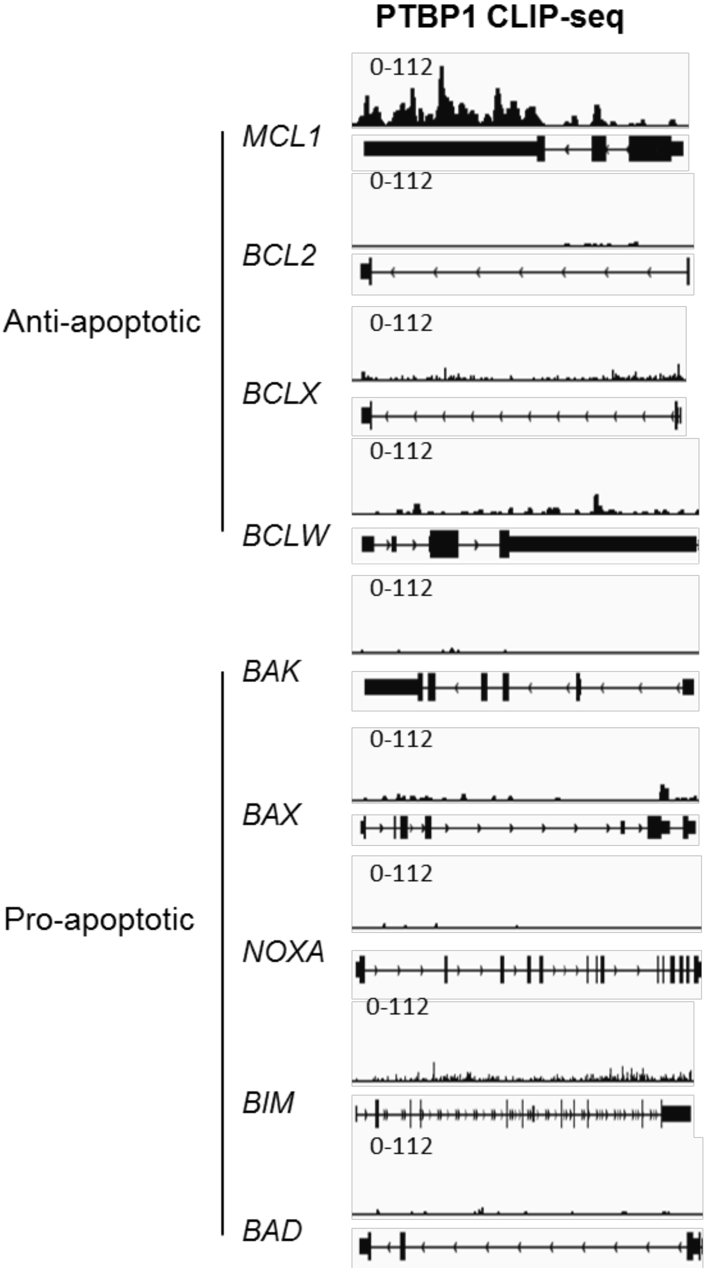


**Supplementary Figure S4**: ***MCL1*** **is the predominant BCL2 family mRNA bound by PTBP1**. CLIP-SEQ data GSM1048186 was analysed to map PTBP1 binding events on different BCL2 family mRNAs. The CLIP-SEQ binding signals were set at the same range (0-112) while the gene lengths were not shown in the same scale.
